# The short and long-term effects of aerobic, strength, or mixed exercise programs on schizophrenia symptomatology

**DOI:** 10.1038/s41598-021-03761-3

**Published:** 2021-12-21

**Authors:** Laura García-Garcés, María Inmaculada Sánchez-López, Sergio Lacamara Cano, Yago Cebolla Meliá, David Marqués-Azcona, Gemma Biviá-Roig, Juan Francisco Lisón, Loreto Peyró-Gregori

**Affiliations:** 1 Department of Nursing and Physiotherapy, Faculty of Health Sciences, Universidad Cardenal Herrera-CEU, CEU Universities, Moncada, Valencia, Spain; 2 State Reference Center for Psychosocial Rehabilitation (CREAP), Valencia, Spain; 3 Department of Sociology of Public Policies of the University of Zaragoza, Zaragoza, Spain; 4 Department of Universal Health and Public Health, Valencia, Spain; 5 Vice Presidency and Ministry of Equality and Inclusive Policies, Valencia, Spain; 6 CV Santos Andrés Santiago and Miguel Foundation, Sueca, Valencia, Spain; 7 Department of Biomedical Sciences, Faculty of Health Sciences, Universidad Cardenal Herrera-CEU, CEU Universities, c/Santiago Ramón y Cajal S/NAlfara del Patriarca, Moncada, 46115 Valencia, Spain; 8 Centre of Networked Biomedical Research in the Physiopathology of Obesity and Nutrition (CIBERobn), CB06/03, Carlos III Health Institute, Madrid, Spain

**Keywords:** Psychiatric disorders, Randomized controlled trials

## Abstract

The purpose of this study was to compare the effects of three different physical exercise programs on the symptomatology, body composition, physical activity, physical fitness, and quality of life of individuals with schizophrenia. A total of 432 patients were assessed for eligibility and 86 were randomized into the aerobic (n = 28), strength (n = 29) or mixed (n = 29) groups. Positive, negative, and general symptoms of psychosis, body mass index (BMI), physical activity (IPAQ-SF), physical fitness (6-min walk test [6MWT] and hand-grip strength [HGS]), and quality of life (WHOQUOL-BREF) were assessed at baseline, post-intervention (16 weeks), and at 10-months. Our results at 16 weeks showed significant improvements in all three groups in the negative, general, and total symptoms with moderate to large effect sizes (*P* < 0.01, η_p_^2^ > 0.11), no change in the BMI, 6MWT or IPAQ-SF, and a significant improvement in the HGS test in the strength and mixed groups (*P* ≤ 0.05, η_p_^2^ > 0.08). Nonetheless, all the improvements had disappeared at 10 months. We concluded that 3 weekly sessions of a moderate to vigorous progressive exercise program for 16 weeks improved the symptomatology of individuals with schizophrenia in all three groups, with no differences between them. However, the effects had declined to baseline levels by the 10-month follow-up, suggesting that exercise interventions should be maintained over time.

## Introduction

Schizophrenia is a major mental health issue worldwide with a lifetime risk of approximately 0.5–1%^1^. It is a chronic disorder characterized by positive symptoms (hallucination, delusion, and thought disorders) and negative symptoms (avolition, apathy, and social dysfunction, among others)^2^. Cognitive impairment is also characteristic of these patients and appears to be related to the negative symptoms^3^, making it one of the leading causes of disability, with severe personal, social, and economic consequences^4^.

A premature mortality gap of between 10 and 20 years is associated with patients with schizophrenia compared to the general population^5^, which is mainly because of premature cardiovascular disease (CVD)^6^. Moreover, genetic factors^7^ and antipsychotic medications^8^ strongly contribute to this high-risk profile, with unhealthy lifestyle habits such as low levels of physical activity (PA)^9^ also playing a particularly prominent role in these increased mortality rates.

Indeed, a recent meta-analysis demonstrated that individuals with schizophrenia engage in low levels of PA^10^ and are less likely to complete PA at a sufficient intensity^11^. Accordingly, these patients tend to have low cardiorespiratory fitness^12^, which has been recognized as a strong and independent predictor of CVDs and all-cause mortality in the general population^13^. In addition, lower participation in PA in these patients correlates with the presence of negative symptoms, the side-effects of antipsychotic medication, social isolation, and other unhealthy lifestyle habits^14^. Moreover, low PA may exacerbate the symptoms of depression, low self-esteem, and impair psychosocial functioning, resulting in lower health-related quality of life^15^.

In contrast, exercise interventions have been consistently proven to prevent CVD^9,16^, alleviate cognitive decline^17^, and improve clinical symptoms, depression, and health-related quality of life^18^ in individuals with schizophrenia. However, there are still significant gaps in the literature regarding which types of exercise might be most effective at improving symptoms, body composition, physical activity, physical fitness, and quality of life in these patients.

Most work studying the effects of PA in patients suffering from schizophrenia has focused on aerobic exercise, and only a few studies have analyzed the effects of mixed (aerobic and strength) programs^19–22^. Of note, as far as we know, only two studies have used isolated strength training programs in schizophrenic patients^23,24^. Additionally, most of the studies have methodological limitations, which makes it difficult to draw any firm conclusions. In this context it is not surprising that several systematic reviews and/or meta-analyses of the effects of PA in patients with schizophrenia have also highlighted issues regarding methodological limitations^18,25,26^ and claim that high-quality data from randomized controlled trials (RCTs) is still lacking.

### Aims of the study

The main objective of the study was to compare the immediate and long-term effects of three different physical exercise programs (strength, aerobic, or mixed) on the symptomatology, body composition, physical activity, physical fitness, and quality of life of patients suffering from schizophrenia.

## Materials and methods

### Study design

This prospective, multi-center, clinical trial (NCT03953664, 16/05/2019) was approved by the Ethics Committee for Biomedical Research at the University Cardenal Herrera in Valencia (Spain) and followed the ethical guidelines set out in the Declaration of Helsinki. All participants agreed to participate and signed the informed consent.

### Eligibility criteria

Eligible participants were all adults aged between 18 and 65 years with a diagnosis of schizophrenia (according to the DSM-5) and who were stable on antipsychotic medication, i.e., who had been using the same dosage for at least 4 weeks prior to inclusion. The exclusion criteria were: (1) a total Positive and Negative Syndrome Scale (PANSS) score of < 58^27^; (2) acute suicidality; (3) patients representing an acute danger to others; (4) the presence of other psychiatric diagnoses or acute psychiatric illnesses; (5) other disorders that could prevent the person from completing the exercise training; (6) participation in similar programs or interventions before enrolment.

### Procedure

This study took place at six psychosocial care centers for people with severe mental illness (SMI), from January 2020 to January 2021. Before the start of the trial, an independent researcher unaware of the study characteristics generated a random sequence using a computerized random number generator; this was concealed from all the other study investigators throughout the entire study period. Randomization was performed with stratification for age, sex, and body mass index (BMI). Upon enrolment in the study and after completing the primary and secondary outcome measures, the participants were randomly assigned either to the aerobic (n = 28), strength (n = 29), or the mixed (n = 29) group.

**Figure 1:**
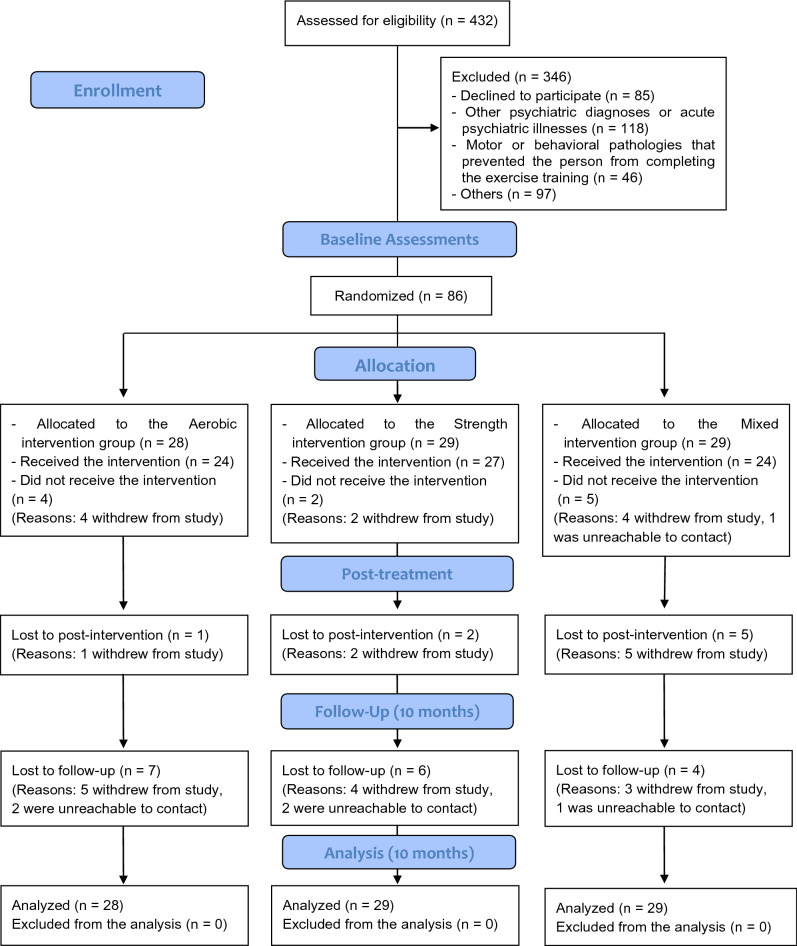
Flow chart.

As shown in the participant flowchart in Fig. 1, all the outcome measures were assessed at baseline, 16 weeks post-baseline (post-intervention), and at a 10-month follow-up. It was impossible to mask the group allocation to the physical therapists or the participants; however, the outcome evaluators and data analysts were blinded to the treatment allocations. To avoid inter-observer variability bias, the measurements in each of the groups were always completed by the same investigator.

### Interventions

Informed consent was obtained from the subject represented in the pictures. The intervention consisted of a total of 48 sessions (3 weekly sessions lasting 1 h each for 16 weeks). To make the comparison fair, the total number of training sessions and their duration were the same for all three training groups. The exercise training was conducted in groups at each psychosocial care center by a professional physical education instructor. Each session began and ended with 10 min of stretching of the major muscle groups.

#### Aerobic training

**Figure 2:**
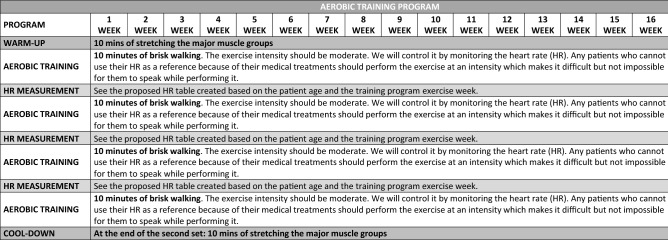
Aerobic training program.

Participants completed 4 series of brisk walking (in the playground of the psychosocial care centers) for 10 min followed by 1 min of recovery. To ensure that the intensity of the exercise progressed from moderate to vigorous, we monitored the heart rate (HR) of each participant using Sport testers (model 610si, Polar, Finland). Progression in exercise intensity was achieved by increasing the participant’s target HR every 2 weeks. Thus, using the formula published by Tanaka et al. to calculate the maximum HR (MHR) (208–0.7 × age)^28^, the intensity of the exercise was progressively increased as follows: weeks 1–2: 55% MHR; weeks 3–4: 58% MHR; weeks 5–6: 61% MHR; weeks 7–8: 64% MHR; weeks 9–10: 67% MHR; weeks 11–12: 70% MHR; weeks 13–14: 73% MHR; and weeks 15–16: 76% MHR (Fig. 2). The physical education instructor continuously checked the HR to adjust the walking speed of each participant in each session.

#### Strength training

**Figure 3:**
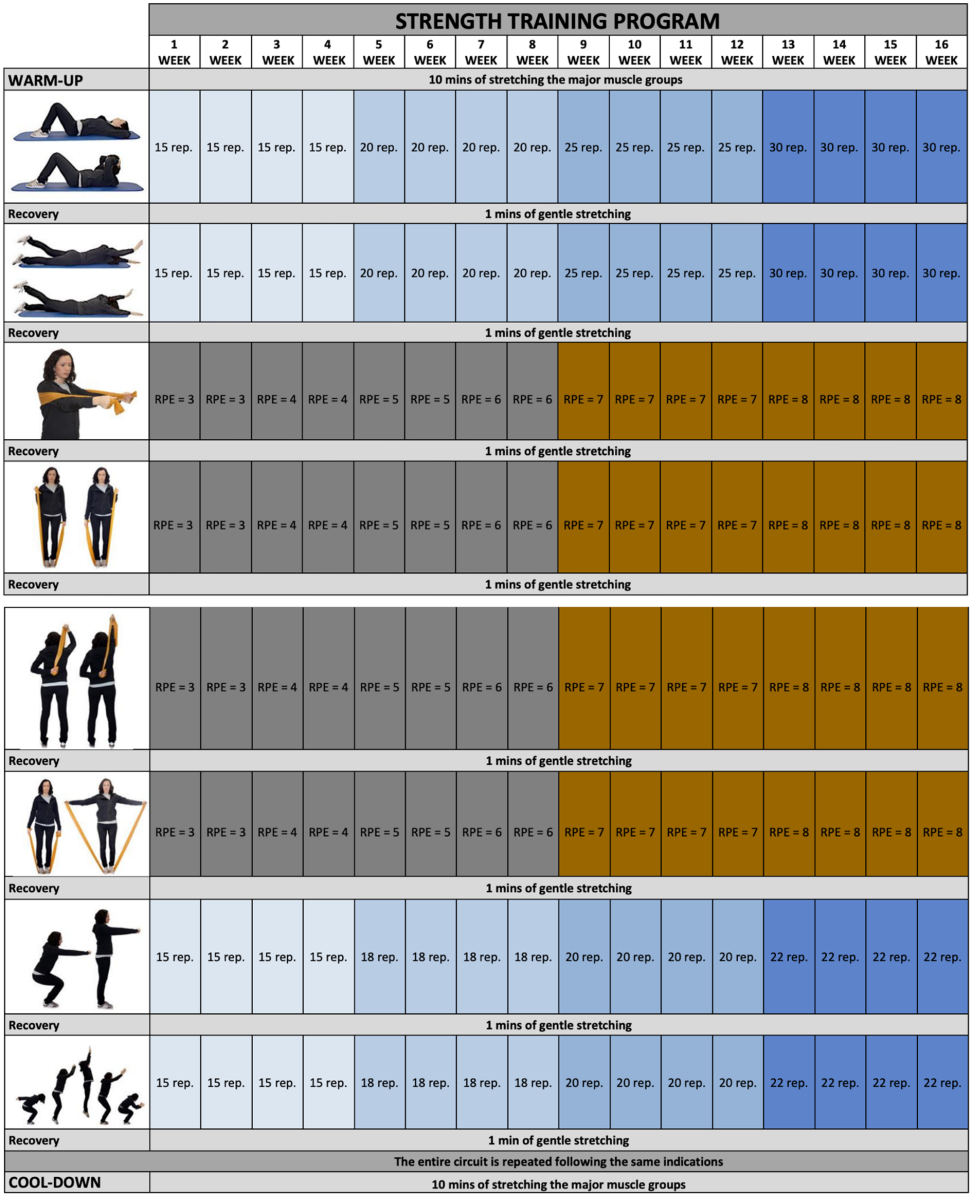
Strength training program.

Participants completed two sets of 8 strength training exercises with 1 min of recovery programmed between each one (Fig. 3; informed consent was obtained from the subject represented in the picture). An elastic resistance band (Thera-band) was used in four of the eight strength exercises. The training intensity increased over the 16 weeks of this intervention; the intensity of exercises completed without an elastic band was amplified by increasing the number of repetitions the participants perform. For exercises performed with an elastic band, the intensity increase was achieved by using the Borg Scale. This scale measures the effort an individual perceives when exercising and creates criteria to adjust the intensity of the programmed exercise. In order to adequately use the Borg scale, the participants learnt to use Theraband resistance bands on the first day and to easily identify, for each exercise, which gripping point on the band was equivalent to an effort that was moderate, intermediate, hard or very hard according to the Borg scale. Figure 3 shows the progression (from 3 to 8) in the rate of perceived exertion (RPE) during the 16 weeks of the program.

#### Mixed training

The main part of each mixed session was divided into two stages. First, like the strength training group, the participants performed 1 set of the same 8 strength exercises with the same intensity and progression, interspersed with 1 min of recovery time per strength exercise. Second, as in the aerobic training group, the participants performed 2 sets of brisk walking for 10 min followed by 1 min of recovery, according to the same exercise intensities and progressions described for the aerobic training group.

All participants were recommended to keep a physically active lifestyle during the 10-month follow-up period.

### Outcome measures

Age, sex, educational level, marital status, employment, institutionalization regime, duration of illness, compliance, and adverse events to the interventions, were all registered. Antipsychotic medication (i.e., amisulpride, aripiprazole, clotiapine, clozapine, haloperidol, levomepromazine, olanzapine, paliperidone palmitate, paliperidone, quetiapine, risperidone, zuclopenthixol, and zuclopenthixol decanoate) was also recorded for each patient and were converted into a daily equivalent dosage of chlorpromazine, according to Gardner et al.^29^. In addition, anticholinergic burden (ACB) was calculated according to Kiesel et al.^30^.

#### Primary outcome

The PANSS is a semi-structured interview which assesses the positive (PANSS-P), negative (PANSS-N), and general (PANSS-G) symptoms of psychosis experienced by patients in the week prior on a 7-point Likert-type scale (from 1, ‘none’, to 7, ‘extreme’)^31^. We separately analyzed the 3 subscales as well as the overall scores (PANSS-T).

#### Secondary outcomes

The BMI was calculated using a SECA® 780 electronic balance scale with a mechanical telescopic stadiometer. Body fat mass (BFM) was determined using a TBF-300A body-fat analyzer. A submaximal exercise test, the 6-min walk test (6MWT), was used to assess the participants’ functional capacity for aerobic exercise. This test has been shown to be a reliable measure of exercise capacity in people with SMI^32^. Hand grip strength (HGS) was assessed using a Jamar hydraulic hand dynamometer, as described elsewhere^33^. The 30-s sit-to-stand (30-s STS) test was used to assess lower limb strength. It consisted of standing-up from a chair and sitting-down again as many times as possible during a 60 s period, with the researcher registering the number of repetitions^34^. PA level was be assessed using the International Physical Activity Questionnaire-Short Form (IPAQ-SF)^35^. Using seven items, this self-reported questionnaire collects data on the patients’ PA in the 7 days prior to the test. The total number of days and minutes of PA were calculated by adding all PA category scores performed over the seven days. Data from the IPAQ-SF were converted into metabolic equivalent minutes per week (METs-min/week), using the formula published by Ainsworth et al.^36^. Specifically, the IPAQ-SF questionnaire records activity at four intensity levels: (1) vigorous activity such as aerobics; (2) moderate activity such as leisure cycling; (3) walking; and (4) sitting. This makes it possible to classify the PA levels of the participants as ‘high’ (> 1500 METs), ‘moderate’ (600–1500 METs), or ‘low’ (< 600 METs). The IPAQ has been validated in 12 countries^37^ and showed adequate psychometric properties and the short version (the IPAQ-SF) has shown acceptable validity in an adult Spanish population^38^. Finally, quality of Life was assessed using the abbreviated WHO Quality of Life Assessment-BREF^39^: This survey comprises 26 items with five Likert-type responses each, and is a standard questionnaire used to measure patient quality of life. It assesses patients under four health domains: physical, psychological, social and environmental. In this study, we will analyze the sum of the four dimensions, with higher scores indicating a better quality of life. This scale has been validated for Spanish and the instrument has a good internal consistency with a Cronbach alpha of 0.88 for the overall scale and a range of 0.70–0.79 for its dimensions^40^.

### Sample size and statistical analysis

The desired sample size was calculated after undertaking a pilot study in 15 participants^41^, which indicated a partial eta^2^ effect size (η_p_^2^) of 0.038 for the primary outcome (PANSS-N). Considering this and using an α value of 0.05 and a desired power of 90%, we used the G*Power (v.3.1.9.2) program^42^ to estimate that a sample of 69 participants would be required. Thus, accounting for potential losses of 25%, we established that the final sample should comprise 86 participants.

Possible confounding factors were assessed by testing baseline differences between the groups using chi-squared (sex, educational level, marital status, employment, and institutionalization regime), Kruskal Wallis (age), and one-way ANOVA (illness duration, BMI, antipsychotic drugs consumption, and ACB scores) tests. ANOVA tests were also used to compare the percentage of attendance rates between the intervention groups.

Two-way mixed ANOVA tests were used to compare how the study interventions affected the primary and secondary outcomes, using time (baseline, post-intervention, and 10-month follow-up) as the within-group factor and group (aerobic, strength, or mixed) as the between-group factor. Analogous ANOVA tests but with two levels in the time factor (baseline and 10-month follow-up) were used to compare how the study interventions affected the consumption of antipsychotic drugs. Bonferroni post-hoc tests were applied following the ANOVAs. The η_p_^2^ effect sizes were calculated such that 0.01 < 0.06, 0.06 < 0.14, and 0.14 or higher, respectively corresponded to a small, medium, and large effect size^43^. The statistical analyses were performed as an intention-to-treat basis using SPSS software (v.18.0) for Windows (IBM Corp., Armonk, NY). All the statistical tests were two-tailed with the critical *P* value for significance set at < 0.05.

## Results

The general characteristics of the study population are shown in Table 1. The statistical analysis did not show between-group differences at baseline for any variable, except for the number of years of illness, for which there were differences between the aerobic and mixed intervention groups. In addition, the percentages of attendance rates did not show statistically significant differences between the aerobic, strength, and mixed intervention groups (88 ± 15%, 88 ± 12%, and 79 ± 15%, respectively; *P* = 0.084). Likewise, the results of two-way mixed ANOVA did not show intra-group or between-group differences in the comparisons of the consumption of antipsychotic drugs. None of the participants reported any adverse events to the interventions.

**Table 1:** Baseline participant characteristics.

Variables	Aerobic group (n = 28), mean (*SD*)	Strength group (n = 29), mean (*SD*)	Mixed group (n = 29), mean (*SD*)
Age (y)	40.9 (10.6)	44.1 (8.0)	43.6 (9.8)
Male (%)	69.6	61.5	58.3
Educational level (% low/intermediate/high)	79/14/7	83/17/0	72/21/7
Marital status (% single)	100	93	100
Employment (% working)	11	17	7
Institutionalization regime (% inpatients)	46.2	35.3	33.3
Duration of illness (y)	13.1 (8.9)	16.7 (8.9)	23.1 (11.4)
Chlorpromazine equivalent treatment dose (mg/day)	873 (831)	981 (666)	702 (690)
Anticholinergic burden score	2.7 (1.0)	2.7 (1.5)	2.8 (1.4)
BMI (kg/m^2^)	29.7 (6.2)	29.8 (4.5)	32.0 (8.0)
BFM (%)	30.2 (9.7)	31.5 (9.3)	34 (10.4)
PANSS-positive (score, 7 to 49 points)	20.3 (4.8)	20.1 (6.6)	20.1 (4.8)
PANSS-negative (score, 7 to 49 points)	25.4 (7.0)	23.1 (6.4)	23.5 (6.1)
PANSS-general (score, 16 to 112 points)	49.8 (7.1)	47.7 (11.4)	48.8 (10.8)
PANSS-total (score, 30 to 210 points)	95.4 (13.9)	90.2 (21.2)	92.3 (18.4)
HGS (kg)	30.4 (12.8)	30.4 (11.0)	25.1 (9.9)
30-s STS (repetitions)	16.1 (5.5)	15.4 (4.3)	16.6 (4.9)
6MWT (meters)	566 (98)	588 (77)	584 (80)
IPAQ-SF (METs)	1904 (2213)	1595 (2138)	1960 (2113)
WHOQUOL-BREF (score, 0 to 100 points)	79.7 (12.3)	81.9 (9.8)	75.2 (10.5)

*BMI* body mass index, *BFM* body fat mass, *PANSS* positive and negative syndrome scale, *HGS* hand-grip strength, *30-s STS* 30-s sit-to-stand test, *6MWT* 6-min walk test, *IPAQ-SF* International Physical Activity Questionnaire-Short Form, *MET* metabolic equivalent of task, *WHOQUOL* World Health Organization Quality of Life.

The results of the Bonferroni post-hoc tests are shown in Table 2. The PANSS-N, PANSS-G, and PANSS-C scores showed statistically significant improvements from baseline to 16 weeks in the 3 groups (*P* < 0.01, η_p_^2^ > 0.11); however, all these significant improvements disappeared at the 10-month follow-up. The PANSS-P scores also improved in the aerobic and strength groups (*P* ≤ 0.005, η_p_^2^ > 0.07), and showed a trend towards improvement in the mixed group (η_p_^2^ > 0.04), but also got worse at the 10-month follow-up. No significant differences were shown in the intra-group comparisons of body composition (BMI and BFM), 30-s STS test, 6MWT, or IPAQ-SF. Of note, there were significant improvements in the HGS test results from baseline to 16 weeks both in the strength and mixed groups (η_p_^2^ > 0.08). However, these improvements also disappeared at the 10-month follow-up. Similarly, the quality of life showed improvements (significantly in the aerobic group) from baseline to 16 weeks that disappeared at the 10-month follow-up. Interestingly, the intra-group comparisons between 16 weeks and the 10-month follow-up did not show statistically significant differences in symptomatology, body composition, 6MWT, or IPAQ-SF in any group. In contrast, the HGS and 30-s STS scores significantly decreased in the strength group, the HGS significantly decreased in the mixed group, and the quality of life significantly decreased in the aerobic group. Finally, no between-group differences were found in any of the primary or secondary outcome comparisons (*P* > 0.05).

**Table 2:** Intragroup comparisons.

Variables	Aerobic group (n = 28)		Strength group (n = 29)		Mixed group (n = 29)		ANOVA effects		
							Time	Group	Time × Group
	Difference (95% CI)	*P* value	Difference (95% CI)	*P* value	Difference (95% CI)	*P* value	F, P value	F, P value	F, P value
**BMI (kg/m**^**2**^**)**									
T1–T0	− 0.3 (− 0.7 to 0.1)	0.142	− 0.1 (− 0.4 to 0.3)	1.000	− 0.1 (− 0.5 to 0.3)	1.000	1.9, 0.16	0.9, 0.41	0.9, 0.46
T2–T0	− 0.3 (− 1.1 to 0.5)	1.000	0.0 (− 0.7 to 0.7)	1.000	− 0.5 (− 1.4 to 0.3)	0.337			
**BFM (%)**									
T1–T0	− 1.1 (− 3.6 to 1.3)	0.754	− 1.0 (− 3.2 to 1.1)	0.739	− 1.9 (− 4.3 to 0.5)	0.174	5.2, 0.007	0.7, 0.50	0.2, 0.95
T2–T0	− 0.1 (− 1.8 to 1.6)	1.000	0.0 (− 1.5 to 1.5)	1.000	− 0.5 (− 2.2 to 1.2)	1.000			
**Chlorpromazine equivalent dose (mg/day)**									
T1–T0	-	-	-	-	-	-	0.1, 0.73	0.4, 0.65	0.3, 0.74
T2–T0	− 33 (− 156 to 90)	0.606	− 36 (− 177 to 105)	0.609	27 (− 102 to 159)	0.666			
**PANSS-positive (score, 7 to 49 points)**									
T1–T0	− 3.2 (− 6.1 to − 0.3)	0.023*	− 2.7 (− 5.4 to 0.0)	0.050*	− 2.2 (− 5.0 to 0.7)	0.206	7.5, 0.001	0.1, 0.89	0.2, 0.89
T2–T0	− 0.5 (− 3.8 to 2.7)	1.000	− 1.5 (− 4.5 to 1.6)	0.708	− 0.3 (− 3.5 to 3.0)	1.000			
**PANSS-negative (score, 7 to 49 points)**									
T1–T0	− 4.2 (− 7.2 to − 1.2)	0.003**	− 3.6 (− 6.4 to − 0.9)	0.006**	− 3.8 (− 6.8 to − 0.7)	0.009**	15.4, < 0.001	1.3, 0.29	0.2, 0.95
T2–T0	− 2.4 (− 5.8 to 0.9)	0.246	− 2.5 (− 5.6 to 0.6)	0.158	− 1.5 (− 4.8 to 1.9)	0.832			
**PANSS-general (score, 16 to 112 points)**									
T1–T0	− 8.7 (− 14.0 to − 3.3)	0.001**	− 7.6 (− 12.6 to − 2.6)	0.001**	− 7.4 (− 12.8 to − 2.0)	0.003**	14.9, < 0.001	0.6, 0.57	0.1, 0.96
T2–T0	− 4.0 (− 11.4 to 3.5)	0.584	− 5.0 (− 11.8 to 1.9)	0.241	− 3.1 (− 10.5 to 4.3)	0.915			
**PANSS-total (score, 30 to 210 points)**									
T1–T0	− 16.1 (− 25.8 to − 6.3)	< 0.001**	− 13.9 (− 22.8 to − 4.8)	0.001**	− 13.4 (− 23.1 to − 3.6)	0.004**	16.3, < 0.001	0.9, 0.40	0.2, 0.94
T2–T0	− 6.9 (− 19 to 5.6)	0.541	− 9.0 (− 20.5 to 2.7)	0.193	− 4.9 (− 17.4 to 7.7)	1.000			
**HGS (kg)**									
T1–T0	− 1.1 (− 4.4 to 2.1)	1.000	3.0 (0.1 to 5.9)	0.039*	3.2 (0.0 to 6.5)	0.050*	3.0, 0.051	0.9, 0.42	3.3, 0.013
T2–T0	0.0 (− 3.0 to 3.0)	1.000	− 1.2 (− 3.8 to 1.5)	0.831	2.0 (− 1.0 to 5.0)	0.323			
**30-s STS (repetitions)**									
T1–T0	0.4 (− 1.5 to 2.2)	1.000	1.5 (− 0.1 to 3.2)	0.083	0.8 (− 1.0 to 2.6)	0.856	6.8, 0.002	0.1, 0.89	0.8, 0.54
T2–T0	− 0.4 (− 2.0 to 1.3)	1.000	− 0.1 (− 1.6 to 1.5)	1.000	− 1.2 (− 2.8 to 0.5)	0.249			
**6MWT (meters)**									
T1–T0	8 (− 42 to 59)	1.000	11 (− 35 to 56)	1.000	− 20 (− 69 to 29)	0.975	0.0, 0.99	0.4, 0.68	0.6, 0.63
T2–T0	12 (− 32 to 56)	1.000	− 4 (− 44 to 36)	1.000	− 6 (− 50 to 37)	1.000			
**IPAQ-SF (METs)**									
T1–T0	49 (− 1423 to 1521)	1.000	672 (− 583 to 1928)	0.573	95 (− 1333 to 1524)	1.000	3.2, 0.044	0.1, 0.93	0.33, 0.85
T2–T0	726 (− 680 to 2131)	0.622	758 (− 441 to 1957)	0.371	920 (− 443 to 2284)	0.300			
**WHOQUOL-BREF (score, 0 to 100 points)**									
T1–T0	8.8 (− 2.1 to 15.5)	0.006**	2.2 (− 3.8 to 8.2)	1.000	6.6 (− 0.1 to 13.2)	0.055	10.3, < 0.001	0.9, 0.41	2.2, 0.07
T2–T0	0.4 (− 5.7 to 6.5)	1.000	− 2 (− 7.5 to 3.5)	1.000	5.1 (− 0.9 to 11.2)	0.125			

Results of the Bonferroni post-hoc test: *P ≤ 0.05; **P ≤ 0.01.

*T0* baseline,* T1* post-intervention, *T2* 10-month follow-up, *BMI* body mass index, *BFM* body fat mass, *PANSS* positive and negative syndrome scale, *HGS* hand-grip strength, *30-s STS* 30-s sit-to-stand test, *6MWT* 6-minute walk test, *IPAQ-SF* International Physical Activity Questionnaire-Short Form, *MET* Metabolic equivalent of task, *WHOQUOL* World Health Organization Quality of Life.

## Discussion

Our results showed significant improvements in the negative, general, and overall score for the symptoms of psychosis at 16 weeks in the aerobic, strength, and mixed training groups, with moderate to large effect sizes. The mean difference in PANSS-T scores nearly reached the level of the minimal clinically important difference, estimated as a 16% to 24% change from baseline^27^. These findings are consistent with several meta-analyses that showed that PA (i.e., aerobic or mixed exercise) significantly reduced the negative^16,18,26^ and total^16,18^ score symptoms for schizophrenia.

To date, only two studies have examined the impact of an isolated strength training program on patients with schizophrenia^23,24^. The pilot study by Heggelund et al.^23^, which consisted of an 8-week intervention (training 3 days per week) with a single exercise (4 × 4 repetitions at 85–90% of the 1 repetition maximum, performed with a leg press apparatus), did not report improvements in the negative symptoms. However, this study only included one exercise type completed for short durations, had a small sample size, and had methodological limitations (it was not randomized, assessment was not blinded, the statistical analysis did not study time × group interactions, and the data were not analyzed on an intention-to treat basis).

The most recent study by Silva et al.^24^ compared the effects of 20 weeks of strength and mixed training programs (2 days per week) on psychotic symptoms. Although the authors did not find between-group differences in the PANSS-N, the intragroup analysis revealed significant improvements in the strength training group. Nevertheless, this study was also limited by some methodological issues (the sample size was small and not calculated a priori, no allocation concealment was reported, and there was no intention-to treat analysis).

Therefore, our results fill a gap in literature because we consistently demonstrated that a strength training program alone was also an effective treatment for the negative and general symptoms of schizophrenia. Indeed, this type of exercise has already obtained good results in diseases such as anxiety and depression^25^ and has been shown to increase cognitive efficiency in older adults^44^ and patients with early dementia^45^.

Our findings showed that there were significant improvements in the positive symptoms at 16 weeks in the aerobic and strength intervention groups (with small to moderate effect sizes) and showed a positive trend in the mixed intervention group. These findings agree with other studies that analyzed the effects of aerobic interventions on positive symptoms. For example, Firth et al.^16^ conducted a meta-analysis of exercise interventions in individuals with schizophrenia and, after performing a sensitivity analysis to exclude low-intensity exercise interventions, reported significant reductions in the PANSS-P scores, with a pooled standardized mean difference (SMD) of − 0.54 (95% CI − 0.95 to − 0.13). The most recent meta-analysis^26^, which included 17 RCTs, conducted a subgroup analysis that differentiated between aerobic interventions (12 RCTs) and non-aerobic interventions (5 RCTs). In this study, aerobic exercise reduced positive symptoms (SMD =  − 0.27; [95% CI − 0.46 to − 0.09]; *P* = 0.004), while non-aerobic interventions did not reduce these symptoms (SMD =  − 0.03; [95% CI − 0.29 to 0.23]; *P* = 0.82). Nonetheless, it must be noted that, rather than training strength, most non-aerobic intervention studies used other types of low-intensity non-aerobic exercises such as yoga^46^.

Contrary to our expectations, in this current work the intragroup analysis failed to show significant improvements at 16 weeks in the mixed training intervention group, and only showed a slight (non-significant) trend. Unlike other mixed intervention studies^21^ that performed a per-protocol analysis and found significant improvements in the positive symptoms, we performed an intention-to-treat data analysis. Although the challenges of this type of analysis include possible loss to follow-up and varying compliance levels, this type of analysis maintains the original group composition achieved by randomization (avoiding potential bias due to exclusion of patients) and pragmatically estimates the benefit of the intervention by estimating how it might perform in a real clinical setting^47^.

In another vein, the fact that our results did not reach statistical significance in the mixed group could perhaps be explained both by the higher attrition rates and the lower compliance of the patients of this group. Hence, even though our 3 exercise interventions were delivered considering 2 major factors known to increase adherence and compliance in schizophrenic patients, i.e., supervision^10^ and a group training setting^16^, the percentages of dropouts were higher and the attendance rates were lower in the mixed intervention group (aerobic = 17.9% and 88%; strength = 13.8% and 88%; and mixed = 34.5% and 79%, respectively).

While the mechanisms by which the different exercise interventions may have influenced the positive and negative symptoms of our patients extends beyond the scope of this study, several mechanisms have been proposed in the scientific literature. The most frequently cited are neuroprotective mechanisms such as decreased inflammation, increased neurogenesis and neuroplasticity via brain-derived neurotrophic factor, and remyelination of white matter tracts^48^.

A recent large-scale meta-analysis confirmed that SMI patients have a significantly increased risk of CVD and CVD-related mortality and that elevated BMI in these patients, often related to antipsychotic drug use, among other factors, requires urgent clinical attention^6^. In particular, weight gain induced by second-generation antipsychotic drugs is a well-documented and prevalent side effect of antipsychotic treatment^49^. Thus, physical exercise is particularly recommended in this population and is included in clinical guidelines for these patients. In agreement with the literature, most of the patients in our study had baseline BMIs corresponding to excess weight (38%) or obesity (46%). In addition, all our patients were on stable antipsychotic medication, with doses that can be considered ‘high’^50^ corresponding to an equivalent of approximately 850 mg/day of chlorpromazine.

Consistent with most previous work^16,20,21,24^, none of our three groups showed significant improvements in body composition at 16 weeks. This agrees with the finding of the meta-analysis by Firth et al.^16^, which included studies with different types of exercise programs, that found no significant post-intervention changes in BMI in these patients. While achieving reductions in BMI and BFM are important exercise goals that should not be abandoned, a more realistic goal may be the attenuation of expected weight gain, to which schizophrenic patients are particularly susceptible^16^. Thus, perhaps our finding of no BMI or BFM gain should be considered noteworthy in itself. Moreover, active but obese individuals have a reduced risk for all-cause and CVD mortality compared to individuals who have a normal BMI but are physically inactive^51^. Therefore, as suggested by Vancampfort et al.^52^, exercise interventions in schizophrenic patients should be primarily developed to improve physical (cardiorespiratory) fitness, with BMI and weight reduction considered as secondary outcomes.

We measured the change in functional exercise capacity using the 6MWT. This test corresponds more to the demands of everyday activities than the maximal oxygen uptake test and is less likely to evoke nervousness or anxiety^53^. Like our results, the studies by Marzolini et al.^20^ and Beebe et al.^54^ which implemented 12-week mixed and 16-week aerobic interventions, respectively, did not find significant improvements in the 6MWT. In contrast, Korman et al.^19^ did report a significant improvement in 6MWT results in their single-arm study (n = 10) with a 10-week mixed intervention. Of these four studies, our study started from the highest basal levels [> 560 m vs. 535^20^, 452^21^, and 430^54^] and therefore had the lowest room for improvement. On the other hand, the systematic review by Bernat et al.^55^ analyzed sixteen intervention studies and found, like our results, that none of them reported a significant increase in the distance walked by adults with schizophrenia.

We measured the levels of physical activity using the IPAQ-SF. In line with the results of the 6MWT, we did not find significant changes in the levels of physical activity in any group—we only found a slight tendency towards improvement-. Although none of the exercise intervention studies discussed here^19–24,54^ assessed physical activity levels (IPAQ-SF) and it is not possible to make direct comparisons, a recent study suggested that the minutes of sitting reported by the IPAQ do not reflect objective sedentary behaviour measurements^56^. These authors concluded that the IPAQ may be unsuitable for the population level assessment of sitting time among individuals with schizophrenia.

As expected, there were only significant intra-group improvements in strength in the strength and mixed training groups. Thus, HGS significantly increased (≥ 3 kg) in both groups. In addition, the 30-s STS test narrowly missed achieving significance in the strength group. Our results agree with other work that also found improvements in strength measured with one-repetition maximum tests after a period of isolated^23,24^ or mixed^20,22^ strength training—i.e., the most recent well-designed randomized controlled trial by Nygård et al.^22^ concluded that twelve weeks of maximal strength training restored patients’ lower extremity force-generating capacity to a level similar to healthy references and improved 30-s STS test performance-. A cross-sectional study concluded that patients with schizophrenia showed lower HGS scores compared to healthy controls, and that HGS scores correlated positively with cognitive functions^57^. A more recent study concluded that higher HGS was associated with greater left and right hippocampal volume and reduced white matter hyperintensities in major depressive disorder (MDD). These authors considered that interventions targeting strength fitness could improve brain health and reduce the neurocognitive abnormalities associated with MDD^58^. Moreover, HGS has been suggested as a risk indicator for cancer mortality^59^ and fatal cardiovascular and all-cause mortality events^60^. Furthermore, isolated strength training has been associated with a lower risk of all-cause mortality, regardless of participation in aerobic PA^61^.

To the best of our knowledge, this current study is the first RCT to compare the long-term effects of 3 different types of exercise programs on the symptomatology, body composition, physical fitness, level of physical activity, and quality of life of patients suffering from schizophrenia. Although none of the 3 programs maintained their significant effects for any variable at the 10-month follow-up, it is remarkable that, without any changes in antipsychotic medication, the variables did not worsen with respect to baseline levels.

This study has limitations that must be considered. First, the patients we enrolled were (a) stable on antipsychotic medication; (b) had PANSS-T scores ≥ 58; and (c) had demonstrated an initial level of motivation to engage in the exercise programs. Therefore, our findings may only be generalizable to individuals with similar characteristics. Second, even though we based our sample size calculations on potential losses of 25%, there were 17 losses to the 10-month follow-up, which meant that our statistical analysis was underpowered by the last time point. Therefore, the long-term effects of these interventions should be interpreted with caution. Finally, the lack of a control group should be considered when interpreting the results. Controls eliminate the alternate explanations of experimental results, especially confounding variables and experimenter bias, enabling the investigator to control for threats to validity.

Despite the availability of comprehensive and evidence-based treatment guidelines, not all patients benefit from standard care (e.g., medication)^62^. While anti-psychotic medication is effective for the positive symptoms of schizophrenia^63^, it is less effective for the treatment of its negative symptoms and cognitive deficits^64^. Unfortunately, these deficits constitute major determinants of poor functioning and disability^3^ and negative symptoms are an important predictor of an unfavorable disease course^26^. Thus, adjunctive therapies like physical exercise that can reduce negative symptoms, cognitive deficits, and improve functional outcomes are vital.

### Perspective

This study concluded that 3 weekly sessions of a moderate to vigorous progressive exercise program for 16 weeks improved the symptomatology of schizophrenic patients in all three groups (aerobic, strength, or mixed), with no differences between them. Additionally, the greatest improvements in strength were achieved with interventions that included strength training exercises. However, their positive effects declined to baseline levels at the 10-month follow-up, suggesting that PA programs should be maintained for longer. Future studies should be deigned to elucidate strategies to keep patients with schizophrenia physically active over time.

## Data Availability

Data available on request from the authors.
